# Green-Sustainable Recovery of Phenolic and Antioxidant Compounds from Industrial Chestnut Shells Using Ultrasound-Assisted Extraction: Optimization and Evaluation of Biological Activities In Vitro

**DOI:** 10.3390/antiox9030267

**Published:** 2020-03-24

**Authors:** Fátima Lameirão, Diana Pinto, Elsa F. Vieira, Andreia F. Peixoto, Cristina Freire, Stefania Sut, Stefano Dall’Acqua, Paulo Costa, Cristina Delerue-Matos, Francisca Rodrigues

**Affiliations:** 1 Polytechnic of Porto—School of Engineering, REQUIMTE/LAQV, Rua Dr. António Bernardino de Almeida, 4249-015 Porto, Portugal; fatinhalameirao@outlook.com (F.L.); diana.pinto@graq.isep.ipp.pt (D.P.); elsa.vieira@graq.isep.ipp.pt (E.F.V.); cmm@isep.ipp.pt (C.D.-M.); 2 REQUIMTE/UCIBIO, MedTech-Laboratory of Pharmaceutical Technology, Department of Drug Sciences, Faculty of Pharmacy, University of Porto, Rua de Jorge Viterbo Ferreira nº. 228, 4050-313 Porto, Portugal; pccosta@ff.up.pt; 3 REQUIMTE/LAQV, Department of Chemistry and Biochemistry, Faculty of Sciences, University of Porto, Rua do Campo Alegre 1021/1055, 4169-007 Porto, Portugal; andreia.peixoto@fc.up.pt (A.F.P.); acfreire@fc.up.pt (C.F.); 4 DAFNAE, Department of Agronomy, Food, Natural Resources, Animals and Environment, University of Padova, Viale dell’Università, 16, Legnaro, PD 35020, Italy; stefania_sut@hotmail.it (S.S.); stefano.dallacqua@unipd.it (S.D.); 5 DSF, Department of Pharmaceutical and Pharmacological Sciences, University of Padova, via Marzolo 5, 35121 Padova, Italy

**Keywords:** *Castanea sativa* shells, ultrasound-assisted extraction (UAE), response surface Methodology, phenolics

## Abstract

Chestnut processing industry generates large amounts of by-products, including leaves, burs and shells that are a source of bioactive compounds. The purpose of this study was to establish an ultrasound-assisted extraction (UAE) of phenolic and antioxidant compounds from industrial chestnut shells. A central composite design (CCD) was conducted to analyze the effects of time (4–46 min) and temperature (34–76 °C) in the antioxidant activity (2,2′-azino-bis(3-ethylbenzothiazoline-6-sulfonic acid (ABTS), 2,2-diphenyl-1-picrylhydrazyl (DPPH), and ferric reducing antioxidant power (FRAP)) and total phenolic compounds (TPC) of chestnut shells extracts. The optimal extraction conditions were obtained at 70 °C for 40 min. The optimal extract was characterized regarding phenolic profile, radical scavenging capacity, and effects on intestinal and dermal cell lines. The optimal extract revealed high amounts of ellagic acid (40.4 µg/mg dw), followed by caffeic acid derivative (15.4 µg/mg dw) and epigallocatechin (15.3 µg/mg dw). Indeed, the extract exhibited the highest scavenging efficiencies against NO● (IC_50_ = 0.1 µg/mL) and HOCl (IC_50_ = 0.7 µg/mL) and did not conducted to a decrease on HaCaT and HFF-1 viability up to 100 μg/mL. Oppositely, a decrease on Caco-2 and HT29-MTX viability was observed. This study suggests that UAE could be a sustainable option to valorize chestnut shells as raw material for different industries.

## 1. Introduction

*Castanea sativa* Mill. is a species belonging to Fagaceae family and genus *Castanea* widely present in Europe. Due to the plurality of applications, not only as food but also as source of wood for wine industry, this species is outstanding, representing an important natural resource for rural populations [1]. Chestnut wood is rich in tannins and extremely resistant. Therefore, it is commonly employed in the manufacture of barrels and casks due to its low porosity and sensitivity to temperature variation. Also, the high levels of aldehydes, phenolic, and volatile compounds are extremely important in the wine aging process [2]. The chestnut production is mainly intended for natural consumption or for production of frozen chestnut. Frozen chestnut production normally occurs for fruits with a caliber inferior to 30 mm. However, semi-processed chestnuts are applied in a huge variety of products, such as jams, purees and flour, among others. The chestnut processing procedure can be divided in three steps: (1) calibration; (2) peeling of the outer shell at high temperatures and (3) removal of the inner shell by water vapor and mechanical processes. Subsequently, the fruits are sorted, manually checked and frozen. During this process, several by-products are generated, mainly shells (inner and outer). Outer and inner shells represent, respectively, 1.5–8.9% and 6.3–10.1% of the total weight of fresh fruit [3]. According to different authors, the main phenolic compounds present in chestnut shells are phenolic acids (ellagic and gallic), flavonoids (rutin, quercetin and apigenin), and tannins [3,4,5]. Phenolic acids, namely ellagic and gallic acid, have been linked to potential health benefits including antioxidant, anticarcinogenic, and anti-inflammatory activities and decrease of cardiovascular disease risk [6,7,8]. Indeed, flavonoids exert numerous biochemical and pharmacological activities, being associated with the increase resistance of blood vessels, treatment of chronic venous insufficiency and improvement of microvascular blood flow [9,10]. According to Morana et al., the inner shell can contain between 2.7% and 5.2% of phenolic compounds [11]. Aires et al. reported that the main phenolic compounds present in aqueous extracts of chestnut shells are gallic acid, ellagic acid, vescalagin, castalagin, epigallocatechin, catechin, and epicatechin [12]. Nevertheless, the bioactive compounds obtained from natural sources depends on the extraction process employed.

Extraction processes are widely used by different industries, such as food, pharmaceutical or cosmetic. In order to reduce production costs and optimize processes, new technologies—such as ultrasound-assisted extraction (UAE)—have been employed to decrease energy consumption and increase the product or process safety/control and quality [13,14]. In fact, conventional techniques (such as Soxhlet extraction or maceration) have long been used to extract bioactive compounds from natural matrices [15]. However, these traditional methods have associated disadvantages, such as low extraction rate, use of large amounts of solvents and high energy consumption [16]. Thus, the implementation of green extraction techniques is a challenge. The UAE is based on the production of longitudinally directed ultrasonic vibrations towards sample (sound waves with frequencies above 20 kHz) capable of causing cavitation [14]. The physical cavitation process generated leads to the formation, growth and collapse of microbubbles within the liquid phase, which facilitate the solvent penetration and improve heat and mass transfer by rupturing cell walls [14,17,18]. Bubble collapse produces extremely high temperatures and pressures, approximately 5000 °C and 100 MPa, respectively [19,20]. Two systems are available for UAE: (1) ultrasound probe and (2) ultrasound bath. Both systems have a transducer that converts mechanical or electrical energy into sound energy (ultrasonic waves) [13]. The ultrasonic bath is a cheap, easy-to-operate device that spans a large number of samples [13]. However, when compared to the probe system, it has a lower cavitation efficiency [14]. On the other hand, the ultrasound probe is generally preferred as it has a direct effect on sample while in the ultrasound bath the effect produced is indirect, as the wavelength of the sound acts in the container before reaching the sample [21]. When compared, the ultrasonic probe has the advantage of producing more energy (allowing a faster extraction), mainly due to the direct immerse in the solution, increasing the contact area with the material and reducing the resistance in the mass transfer [14]. In contrast to conventional extractions that are not suitable to be implemented in a large-scale, an upscale of UAE represents a feasible technological alternative for industrial applications. The interest for this eco-friendly technique has been increasing in the last decades with some companies using it to produce extracts from natural sources [13,22,23]. UAE has emerged as an efficient, rapid, energy and time-saving, and clean extraction methodology, providing a higher recovery of bioactive compounds using low amounts of solvent [13,22,23]. As an example, Pan et al. extracted polyphenols from pomegranate peels using UAE and described a reduction of 87% of extraction time and an increase of 22% in the antioxidant activity compared to the extracts obtained by maceration [24]. Pingret et al. performed lab- and pilot-scale UAE extraction of polyphenols from apple pomace and reported a stronger antioxidant activity and a total phenolic content 30% higher than the one determined for conventional extraction [25]. To the best of our knowledge, no studies report the use of UAE for the extraction of bioactive compounds from *C. sativa* shells (CSS). This paper was designed to extract active ingredients from CSS using UAE as eco-friendly technology in order to valorize this by-product and aware industry for a new potential ingredient. The extraction was optimized by response surface methodology (RSM) with view to obtain an extract with high antioxidant activity. Indeed, the phenolic profile was assessed to understand individual phenolic compounds responsible for the activity, as well as radical scavenging activity and in vitro effects on different intestinal and skin cells.

## 2. Materials and Methods

### 2.1. Chemicals

All chemical reagents, solvents and standards were of analytical reagent grade and obtained from Sigma-Aldrich (St. Louis, MO, USA). Standards used for the quantification or for the identification of compounds, namely by LC-MS, were purchased from Sigma-Aldrich (Steinhemin, Germany) and Extrasynthese (Genay Cedex, France).

### 2.2. Sample

*Castanea sativa* shells were kindly supplied by Sortegel, located in Sortes (Bragança, Portugal, latitude 41°42’18.6” N and longitude 6°48’36.6” W), in October 2018. Shells were dehydrated (Excalibur Food Dehydrator, Sacramento, CA, USA) at 41 °C for 24 h and grounded in a miller (Ultra Centrifugal Mill ZM 200, Retsch, Germany) to particles sizes of 1 mm. Afterwards, samples were thoroughly mixed and stored at room temperature (20 °C) and under light-free conditions until extraction.

### 2.3. Ultrasound-Assisted Extraction (UAE) of Bioactive Compounds from C. sativa Shells

The UAE was carried out using an ultrasonic device (Sonic Vibracell, model VC 750, Newtown, CT, USA), comprising a 13 mm diameter tip with amplitude, temperature and time controller. The amplitude employed was 50%. The powdered samples (5 g) were extracted with 100 mL of water into the ultrasonic device at different times and temperatures, as defined by the RSM design. After ultrasonic extraction, the extracts were filtered through Whatman n° 1 paper, centrifuged (Sigma 3-30KS, Sigma, Osterode am Harz, Germany) at 16,000× *g* for 10 min and frozen at –80 °C for subsequent lyophilization (Telstar, model Cryodos –80, Barcelona, Spain). Samples were stored at 4 °C until analysis. For the subsequent analyses, the final residue was dissolved in water.

### 2.4. Experimental Design, Modelling, and Optimization of Antioxidant Conditions

The RSM is employed to modulate and analyze challenges in which the responses studied (dependent variables) are influenced by independent variables. The RSM was applied to optimize the UAE, with the purpose of selecting favorable extraction conditions and maximize the efficiencies to extract bioactive compounds. A central composite design (CCD) was built for optimization of the best extraction conditions to obtain the highest antioxidant activity and phenolic composition. The CCD variables under analysis were time (*X*_1_, 4, 10, 25, 40, and 46 min) and temperature (*X*_2_, 34, 40, 55, 70, and 76 °C). A total of 13 experiments were randomly performed (Table 1). A complete 22-factorial design characterizes CCD that comprises cubic points, with four axial points at a distance of α = 1.414 from the design center, and five center points. The Total Phenolic Content (TPC; *Y*_1_) and the antioxidant activity analyzed by ferric reducing antioxidant power (FRAP) (*Y*_2_), 2,2-diphenyl-1-picrylhydrazyl (DPPH) (*Y*_3_), and 2,2′-azino-bis(3-ethylbenzothiazoline-6-sulfonic acid (ABTS) (*Y*_4_) assays were the responses studied. The analysis of the regression equations, response surfaces, and contour plots using the predictive equations of RSM allows the determination of the optimal values of Y responses. A set of experiments using the critical values optimized were carried out to evaluate the accuracy of the models. In this way, a t-test was employed to compare the responses under optimized conditions with those predicted by CCD model.

#### 2.4.1. ABTS Radical Scavenging Activity Assay

The ABTS radical scavenging activity of extracts was evaluated according to Re et al. [26], with minor modifications. A calibration curve was obtained using known concentrations of ascorbic acid as standard (linearity range: 5–100 µg/mL, *R*^2^ > 0.993). The results were expressed in terms of IC_50_ (extract concentration providing 50% of radical scavenging activity).

#### 2.4.2. Ferric Reducing Antioxidant Power (FRAP) Assay

Ferric reducing antioxidant power was determined according to Benzie and Strain [27], with minor modifications. Results were expressed in terms of IC_50_ (extract concentration providing 50% of radical scavenging activity).

#### 2.4.3. DPPH Free Radical Scavenging Assay

DPPH free radical scavenging assay was performed according to Barros et al. [28], with minor modifications. The results were expressed in terms of IC_50_ (extract concentration providing 50% of radical scavenging activity).

#### 2.4.4. Total Phenolic Content (TPC)

The total phenolic content (TPC) quantification was performed based on the Folin–Ciocalteu procedure [29], with minor modifications, being expressed as mg of gallic acid equivalents (GAE) per gram of plant material on dry weight (dw).

### 2.5. Characterization of the Optimal CSS Extract

#### 2.5.1. ROS and RNS Scavenging Capacity Assays

A Synergy HT Microplate Reader (BioTek Instruments, Inc., Winooski, VT, USA), capable to measure fluorescence, chemiluminescence and UV/Vis and equipped with a thermostat, was used to evaluate the reactive oxygen and nitrogen species (ROS and RNS, respectively) scavenging capacity. To obtain the values of IC_50_, the curves of percentage of inhibition versus extract concentration were analyzed. The superoxide anion radical (O_2_●^−^), hypochlorous acid (HOCl), peroxyl radical (ROO●), and nitric oxide (NO●) scavenging capacity assays were performed according to Gomes et al. [30]. Catechin and gallic acid were used as positive controls in the scavenging assays. Samples were prepared as previously described by Marangi et al. [31].

#### 2.5.2. Identification of Phenolic Compounds by Qualitative and Quantitative Analysis by ^1^H NMR and LC-UV-MS

NMR analysis were obtained on a Bruker Avance III spectrometer operating at 400 MHz. Standard Bruker sequences were used for the acquisition of mono and dimensional experiments. Measurements of 90° pulse length/power and relaxation delay were performed for each sample. The LC-DAD-MS analysis was performed using two different chromatographic approaches: (i) one using a hydrophilic interaction liquid chromatography (HILIC), on a Tosohas amide 80 (2.1 × 150 mm, 3.5 µm) column and (ii) another employing a reverse phase (C18) stationary phase on a Phenomenex RP-Polar (3 × 150 mm, 4 µm) column. For the HILIC, water 1% formic acid (A) and acetonitrile (B) were used as eluents. Gradient was as follow: start with 99% of B, in 20 min 80% B, the composition was isocratic up to 25 min, then in 45 min was changed to 35% B and in 51 min 20% A. The flow rate was 200 µL/min. For the second chromatographic approach, the eluents were as follows: water 1% formic acid (A) and methanol (B), gradient start with 90% of A and in 20 min reach 90% B. The flow rate was 0.4 mL/min. At the end of the HPLC column, a ”T” connector splits the flow rate half to DAD, half to mass spectrometer and half to fluorimetric (FLX) or diode array detector (DAD). DAD chromatograms were detected at different wavelengths (254, 280, 350 nm) and the UV-VIS spectra were acquired in the range of 200–600 nm. Fluorescence chromatograms were obtained using excitation at 230 nm and emission at 320 nm. MS spectra were recorded in negative or in positive ion mode in 50–2000 Da range, using an ESI ion source. The Turbo Data Depending Scanning (TDDS) function of the mass spectrometer was used to study the fragmentation schemes of the prevalent ions detected. The compounds identification was based on the fragmentation spectra, as well as, in the comparison of the mass spectrometric data with literature and injection of reference compounds when available. For quantification purposes, calibration curves of the reference compounds, namely chlorogenic acid (5–100 µg/mL) and rutin (8–80 µg/mL), were obtained by diluting stock standards solution. Linear regressions were as follows: (i) rutin (280 nm), *y* = 11.101*x* + 0.014 (*R*^2^ = 0.999); (ii) chlorogenic acid (330 nm) *y* = 17.11*x* – 0.121 (*R*^2^ = 0.999). For Procyanidin quantification, Procyanidin B1 was used as reference and FLX (ex 230; emission 320) was used for quantification purposes. The calibration curve was obtained in the range 5–50 ug/mL and the curve equation was *y* = 0.98*x* + 0.002 (*R*^2^ = 0.992).

#### 2.5.3. Cells Viability Assay

The 3-(4,5-dimethylthiazol-2-yl)-2,5-diphenyltetrazolium bromide (MTT) assay was performed to evaluate the effect of the optimal CSS extract on intestinal and skin cell lines. Briefly, cells were incubated during 24 h with fresh medium in the absence or presence of extracts (0.1, 1, 10, 100, and 1000 μg/mL) dissolved in cell culture medium. Four cell lines were employed: (i) Caco-2 clone type C2BBe1 cells, provided by American Type Culture Collection (ATCC, Manassas, VI, USA); (ii) HT29-MTX cell line, obtained from Dr. T. Lesuffleur (INSERMU178, Villejuif, France); (iii) human immortalized non-tumorigenic keratinocytes cell line HaCaT, acquired from CLS Cell Lines Service, Germany; and (iv) human foreskin fibroblasts (HFF-1), purchased from ATCC (ATCC Number SCRC-1041; ATCC, Manassas, VA, USA). Passage 81–84, 35–38, 21–23, and 83–85 from Caco-2, MTX-HT29, HaCaT and HFF-1 were, respectively, used for the MTT assay. Cells were grown according to the methodology described by de Francisco et al. [32].

### 2.6. Statistical Analysis

Results were presented as mean ± standard deviation of at least triplicate experiments. Design Expert trial version 7 (Stat-Ease Inc., Minneapolis, MN, USA) was used to the determination of the regression equations, analysis of the response surface and contour plots, and statistical analysis of the experimental design. IBM SPSS Statistics 24.0 software (SPSS Inc., Chicago, IL, USA) was used to perform the analysis of the data. One-way ANOVA was applied to investigate the differences between samples for all assays and post hoc comparisons were performed with Tukey’s HSD test. A denoting significance was accepted for *p* < 0.05 in all cases. A correlation study was also performed, being selected the Pearson’s correlation coefficient ‘R’. In ROS and RNS assays, the GraphPad Prism 7 software (GraphPad, La Jolla, CA, USA) was used to calculate the IC_50_ values based on the curves of inhibition percentage versus antioxidant concentration.

## 3. Results and Discussion

The extractive process is an essential step in the recovery and purification of bioactive compounds (such as phenolic compounds) from plant matrices [33]. The extraction efficiency depends on several parameters, such as temperature, time, solvent polarity, pH, among others [34]. The RSM is a useful statistical toll for assessing the effect and interaction of many of these variables and finding the variables combinations that will produce the optimal response [35]. In this work, the variables under study were temperature and time; UAE experimental conditions, as well as the predicted and experimental values of TPC, FRAP, DPPH and ABTS assays of *C. sativa* shell extracts are presented in Table 1. The yield value of each extraction is also shown in Table 1, ranging from 7.3% (extraction 1, 40 °C, 10 min) to 16.1% (extraction 4, 70 °C, 40 min). Regarding the evaluated activities of the extracts, TPC ranged from 255.8 mg GAE/g dw (extraction 5, 55 °C, 4 min) to 418.0 mg GAE / g dw (extraction 7, 34 °C, 25 min); FRAP varied between 28.7 µg/mL (extraction 7, 34 °C, 25 min) and 45.3 µg/mL (extraction 5, 55 °C, 4 min); DPPH ranged from 43.7 µg/mL (extraction 7, 34 °C, 25 min) to 63.7 µg/mL (extraction 5, 55 °C, 4 min); ABTS ranged from 50.5 µg/mL (extraction 5, 55 °C, 4 min) to 88.0 µg/mL (extraction 12, 55 °C, 25 min). The results of variance analysis (ANOVA) allow the evaluation of the adequacy and significance of the models through Fisher’s *F* test and are presented in Table 2.

The independent variable *X*_1_ showed a significant effect on the TPC (*p* < 0.01) and FRAP (*p* < 0.05) response, while the independent variable *X*_2_ had no significant effect on any response. The quadratic term for *X*_1_ exhibited a significant effect on TPC (*Y*_1_) and antioxidant activity through FRAP (*Y*_2_) and ABTS (*Y*_4_) assays, while the quadratic term for *X*_2_ showed a significant effect on FRAP (*Y*_2_) and ABTS (*Y*_4_) responses. The *R*^2^ is useful to verify the adequacy of the model. The values showed that the models explained 84.90%, 85.42%, 75.74%, and 74.76% of the variation of *X*_1_ and *X*_2_ for the respective TPC, FRAP, DPPH, and ABTS responses (Table 2). For all response surface models, the “lack of fit” was not significant (*p* > 0.05) and the Ratio was greater than 4 (as desired), indicating an adequate signal to noise ratio. All these indicators confirmed the adequacy of the model to represent the experimental data and to predict the four parameters analyzed. Response Equations (1)–(4) show the dependence of TPC (*Y*_1_), FRAP (*Y*_2_), DPPH (*Y*_3_), and ABTS (*Y*_4_) on time (*X*_1_) and temperature (*X*_2_) and were determined by regression analysis:

(1) $$\mathrm{TPC}=456.89+9.00xX_{1}-9.23xX_{2}-0.02x\;X_{1}xX_{2}-0.12x\;X_{1}^{2}+0.09X_{2}^{2}$$

(2) $$\mathrm{FRAP}=6.81-0.66xX_{1}+1.50xX_{2}-0.001x\;X_{1}xX_{2}+0.01x\;X_{1}^{2}-0.01X_{2}^{2}$$

(3) $$\mathrm{DPPH}=-27.68+0.42xX_{1}-3.07xX_{2}-0.02x\;X_{1}xX_{2}+0.008x\;X_{1}^{2}-0.02X_{2}^{2}$$

(4) $$\mathrm{ABTS}=-43.59+2.78xX_{1}-3.29xX_{2}-0.002x\;X_{1}xX_{2}-0.05xX_{1}^{2}-0.03X_{2}^{2}$$

**Figure 1:**
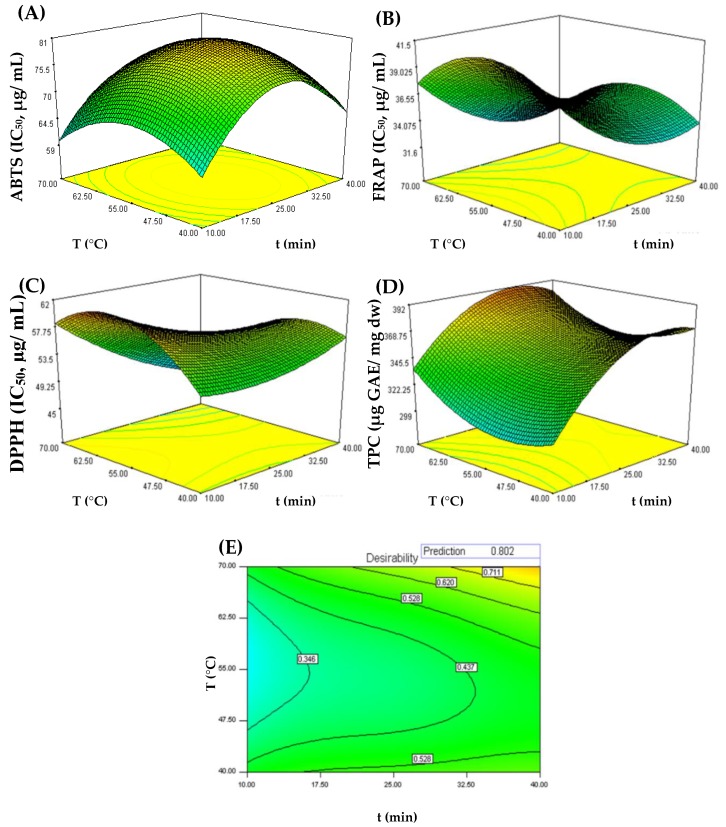
Response surface plots for interaction effects of time (min) and temperature on ABTS (**A**), FRAP (**B**), DPPH (**C**), and TPC (**D**) extraction and on the desirability index for combined responses of UAE-CSS extracts (**E**). The optimal points were identified on the response surfaces.

The RSM model was used to generate 3D contour graphs to represent the relationship between the independent variables (time and temperature) and the dependent variables (ABTS, FRAP, DPPH and TPC). Figure 1 shows the response surface graphs of antioxidant activity assessed by three assays (ABTS, DPPH and FRAP), as well as TPC as a function of time and extraction temperature.

As shown in Figure 1A–D, temperature and extraction time influence ABTS, DPPH, FRAP and TPC assay responses, respectively. However, the TPC response showed a distinct variation with respect to the antioxidant activity assessed by ABTS, DPPH, and FRAP assays. Effectively, a higher antioxidant activity was found (DPPH = 45.5 µg/mL; ABTS = 59.9 µg/mL; FRAP = 31.7 µg/mL) when UAE conditions were 70 °C and, respectively, 40, 10, and 34 min, suggesting that under these extraction conditions more compounds with higher antioxidant activity are extracted or probably formed. The desirability for these assays was greater than 0.7492. In contrast, there was a decrease in the antioxidant activity when the extraction temperature was 55 °C. In the case of the TPC, it is possible to observe an increase in polyphenol content as the temperature and extraction time increased, reaching a maximum response (391.6 mg GAE/g dw) at the extractive conditions of 70 °C and 31 min; the desirability was 0.8925. These results are in line with Gironi and Piemonte [36], who reported that the use of high temperatures (50–80 °C) increases the extraction of bioactive compounds from plant matrices.

According to the desirability graph (Figure 1E), optimal UAE conditions to simultaneously maximize antioxidant activity and TPC were 40 min at 70 °C (*R*^2^ = 0.8024). As shown in Table 3, the experimental value for the four responses was similar to the value predicted by the model (*p* < 0.05), showing once again the effectiveness of RSM for the optimization of antioxidant compounds and polyphenols extraction from chestnut shells using UAE.

Generally, extracts containing high amounts of polyphenols also exhibit high antioxidant activity [37]. According to Table 3, the TPC was 393.1 mg GAE/g dw. Previously, Barreira et al. evaluated the antioxidant properties of *C. sativa* shell extract using water at 100 °C for 30 min as extraction solvent (with a solid/liquid ratio of 5 g/50 mL) [38]. The TPC determined for outer and inner shells were, respectively, 510 and 475 mg GAE/g dw, being higher than the TPC of the present study. These differences could be explained by the use of a higher solid/liquid ratio (5 g/50 mL) [38]. In another study, Nazzaro et al. evaluated the TPC of *C. sativa* shells using water at room temperature as extraction solvent during 5 days [39]. The TPC verified was 333.2 mg GAE/g dw [39]. According to Squillaci et al., *C. sativa* shells extract, when subjected to water extraction at 100 °C for 60 min, contains a high content of phenolic compounds (206 mg GAE/g dw) [1]. This value, obtained by a conventional technique using a similar solid/liquid ratio (5 g/100 mL), is lower than the one obtained in the present study. Rodrigues et al. evaluated the TPC of *C. sativa* shell (extracted with ethanol: water (1:1) at 50 °C for 30 min (with a solid/liquid ratio of 5 g/100 mL) using a conventional technique [40]. The results obtained ranged from 241.9 to 796.8 GAE/g dw for the Portuguese regions of Minho and Trás os-Montes, respectively [40]. Comparing the value obtained in the present study (393.1 mg GAE/g dw) with the values reported by different authors for the same by-product, it is possible to conclude that UAE not only improved the extraction time but also decreased the temperature used, allowing to obtain similar and higher results.

According to Table 3, the IC_50_ determined for the DPPH assay was 44.1 µg/mL, being higher than the antioxidant activity obtained for Trolox (IC_50_ = 51.6 µg/mL). Almeida et al. reported an IC_50_ = 17.7 µg/mL for *C. sativa* leaves extracted with water (during 20 min at 40 °C and 500 rpm) [41]. Similarly, Pinto et al. determined the antioxidant activity of hydroalcoholic extracts of *C. sativa* burs from different regions of Portugal (prepared at 40 °C during 30 min), achieving a slightly higher DPPH● scavenging ability (IC_50_ = 38.7 µg/mL) for Minho samples [42]. In fact, polyphenols are secondary metabolites that perform a key role in the defense and survival of plants, having important functions in their adaptation to biotic and abiotic conditions [43,44]. In this sense, plants produce polyphenols as a defense mechanism against the attacks on plant tissues or in a stressful environment (e.g., unfavorable temperature, light and pH conditions) [44]. The environmental conditions, namely light, soil nutrients, temperature or water availability can effectively influence the phenolic composition and concentration in plants and its derivatives [43]. Also, the type and concentration of polyphenols varies depending on the plant tissue and its stage of development [43]. The high antioxidant activity of chestnut shells and leaves extracts obtained at lower temperatures is probably related to the high amount of polyphenols as a consequence of the exposure to *stress* conditions, initiating the plants’ defense mechanism [40,42,43]. Comparing the results obtained with those reported for walnut shells (IC_50_ = 0.35 mg/mL), hazelnut skins (IC_50_ = 1.0 mg/mL) and almond shells (IC_50_ = 193.6 µg/mL), the antioxidant activity is considerably higher [45,46,47].

According to Table 3, the IC_50_ determined for the ABTS assay was 65.4 µg/mL while for ascorbic acid (data not shown in the table), used as reference, an IC_50_ = 42.0 µg/mL was achieved. Fernández-Agulló et al. analyzed the antioxidant activity of *C. sativa* shells and burs extracted using conventional methodologies, with different solvents, at 75 °C during 60 to 120 min [48]. According to the authors, the IC_50_ determined for the ABTS assay on chestnut shells was 0.3 mg/mL (using water as a solvent), while for bur the result was 0.5 mg/mL (with water: ethanol (1:1)) [48]. Thus, the antioxidant activity determined in the present study is higher.

Finally, an IC_50_ = 32.0 µg/mL was obtained for the FRAP assay. Dinis et al. analyzed the antioxidant activity of chestnut from different ecotypes of the Trás-os-Montes region (Portugal) extracted with 50% ethanol during 1 h [49]. The IC_50_ ranged between 6.6 µg/mL and 14.6 µg/mL, being the antioxidant activity higher than the one obtained in the present study, which could be explained by weather conditions and extraction solvents used [49]. However, when the comparison is made with other food by-products, such as *Q. cerris* seeds (IC_50_ = 203.1 µg/mL) or olive leaves (IC_50_ = 180 µg/mL), the chestnut shell presented a higher antioxidant activity [50,51].

Overall, the high antioxidant activity of extracts, proved by ABTS, DPPH and FRAP assays, is probably related with the TPC. Therefore, it is possible to conclude that UAE allowed a high recovery of polyphenols with remarkable antioxidant properties, reducing the extraction time and ensuring low environmental impacts. In particular, most of the studies about chestnut shells used high temperatures and/or long extraction times for polyphenols recovery, which comprises a disadvantage to an industrial application since the extraction process becomes more expensive and increases the manufacturing costs [1,38,48]. In addition, water was efficiently used in the present study as a green and cheaper solvent, in contrast with previous studies cited that employed ethanol [40,48,49].

### 3.1. ROS and RNS Scavenging Assays

Reactive species are one of the main causes of oxidative *stress*, being responsible for the aging process [35,52]. The overproduction of ROS may have negative impact on essential cells molecules, including lipids, proteins and DNA. However, due to their antioxidant capacity and radical scavenging activity, phenolic compounds may attenuate the effects of this process, preventing the biochemical consequences of oxidation [30,35]. Table 4 summarizes the in vitro scavenging capacity of *C. sativa* shells extract and the positive controls used.

Similarly to previous studies, different assays were employed to investigate the effectiveness of CSS extract to scavenge reactive species produced in human body (ROS and RNS) and to estimate its potential use as active ingredient with benefits in the prevention of oxidative stress-mediated disorders [31,35,50]. The scavenging capacity of extracts differs for each reactive species, which supports the need to measure this activity for different reactive species [31,32,50]. Among the ROS and RNS studied, the highest scavenging efficiencies of the optimal CSS extract were achieved for NO● (IC_50_ = 0.1 µg/mL) and HOCl (IC_50_ = 0.7 µg/mL).

Superoxide radical formation is the first step for the generation of oxygen species, being considered the starting point of oxidative stress [53,54]. According to Table 4, the optimal extract presented an IC_50_ = 14.1 µg/mL for the scavenging of this species, being lower than the result obtained for catechin (IC_50_ = 49.0 µg/mL). Almeida et al. and Reinoso et al. evaluated the protective effect of *C. sativa* leaves extract with respect to ROS uptake capacity, obtaining similar results for the superoxide radical (respectively an IC_50_ = 13.6 μg/mL and IC_50_ = 15.1 μg/mL) [4,52].

In what concerns to the uptake of peroxyl radical, the result present in Table 4 (0.3 µmol TE/mg dw) is lower than the ones reported by Almeida et al. (1.2 µmol TE/mg dw) and Reinoso et al. (1.1 µmol TE/mg dw) for chestnut leaves [4,52]. To the best of our knowledge, this study reports for the first time the scavenging activity of chestnut shells against this reactive oxygen species.

Indeed, the optimal extract had a good scavenge capacity of hypochlorous acid, achieving a low IC_50_ (0.7 µg/mL) that is between the two positive controls used, catechin (0.2 µg/mL) and gallic acid (1.3 µg/mL). In a recent study, Marangi et al. evaluated an aqueous extract of *A. arguta* leaves obtaining an IC_50_ = 1.7 µg/mL [31]; the scavenging activity of this species was lower than the optimal extract developed in the present work. The scavenging power of the optimal extract against NO● (IC_50_ = 0.1 µg/mL) was also higher than *C. sativa* leaves extract (IC_50_ = 3.1 µg/mL and IC_50_ = 7.2 µg/mL, respectively) [4,52]. In addition, a lower quenching ability was obtained for *A. arguta* leaves extract (IC_50_ = 3.80 µg/mL) [31].

**Figure 2:**
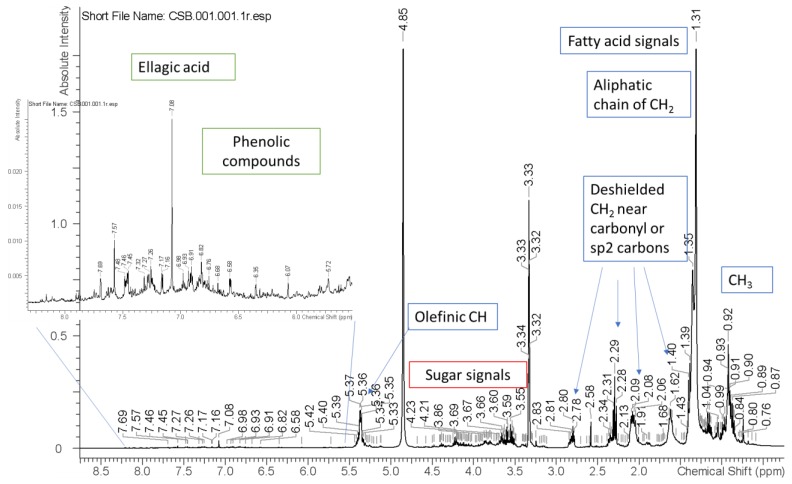
^1^H NMR of the optimal extract recorded in D_2_O.

Considering the previous results, the optimal extract prepared by UAE showed promising results concerning the quenching ability against the ROS and RNS probably due to the phenolic composition. In particular, the presence of ellagic acid, catechin/epicatechin, epigallocatechin and a caffeic acid derivative, whose scavenging power has been described by several authors [55,56], may contribute to this activity (Figure 2; Table 5).

### 3.2. ^1^H NMR and LC-MS Analysis

The multi-technique approach ^1^H NMR and liquid chromatography coupled with diode array, fluorescence and Mass spectrometry, were used to establish the phytochemical composition of the optimal extract. From ^1^H NMR spectrum it is possible to observe characteristic signals of fatty acid derivatives, sugar residues and phenolic compounds. For these latter compounds, the significant signals were the singlet at δ 7.15 ppm that appear to be correlated in the HSQC spectrum to an aromatic carbon resonance at δ 108.6 ppm, supporting the presence of ellagic acid derivatives. Furthermore, several signals in the aromatic part presenting not resolved peaks in the region of δ 6.7–6.0 ppm and 6.5 ppm suggest the presence of high polymeric phenolics as procyanidins and flavonols. Other signals are also present as singlets or doublets in the region of δ 5–5.5 ppm, indicating the presence of sugar, or ester linked OCH of sugars.

Further data were obtained using LC-DAD-ESI-MS^n^ in the negative ionization mode (Table 5), showing a limited number of peaks with significant UV absorption and MS spectra. C-18 and HILIC separations were obtained. The most efficient separation was achieved on HILIC column and an exemplificative chromatogram is reported in Figure 2.

The results of C-18 analysis allowed the identification of caffeic acid derivatives in the first part of the chromatogram and ellagic acid as main constituent. Other minor species were detected and showed significant mass spectra, but it was not possible to assess their tentative structure due to the low amount. The observation of the NMR spectra suggested the presence of low amounts of procyanidins.

According to Table 5, the most abundant compounds are ellagic acid (40.4 µg/mg), epigallocatechin (15.3 µg/mg) and caffeic acid derivative (15.4 µg/mg). These results are considerably higher than the ones reported by Aires et al. for chestnut peel extracts prepared by conventional extraction at 85 °C using different solvents (water, Na_2_SO_3_ and NaOH at different concentrations of 1, 2, 4, and 8% in water) and extraction times (30, 60, and 120, 240, 480, and 960 min) [12]. According to the authors, the major phenolic compounds were gallic acid (7.9–584.9 μg/g dw), ellagic acid (47.6–3542.6 μg/g dw), vescalagin (67.5–109.4 μg/g dw), castalagin (49.6–100.4 μg/g dw), epigallocatechin (13.6–213.4 μg/g dw), catechin (151.1–295.9 μg/g dw), and epicatechin (9.0–66.8 μg/g dw). Ellagic acid (3542.6 μg/g dw) was the major individual polyphenol quantified, being extracted with 1% NaOH during 120 min [12]. Furthermore, the total amounts of polyphenols determined by Aires et al. ranged from 72.5 to 4033.3 μg/g dw, being significantly lower than the one obtained in the present study (97.8 μg/mg) [12]. A possible explanation for these differences might be the use of different extraction techniques as well as extraction conditions and solvents.

On another study, Comandini et al. described the presence of gallic acid, ellagic acid, castalin, castalagin, 1-*O*-galloyl castalagin, vescalin, and vescalagin after extraction of chestnut bark at ambient temperature during 30 min and sonicated for 30 min in an ultrasonic bath operating at a frequency of 35 kHz [57]. According to Squillaci et al., the aqueous extracts of the chestnut inner and outer shell, as well as the exclusive inner shell, contain a high content of phenolic compounds, with gallic acid being the most abundant (63.5 and 29.6 mg/g dw for inner and outer shell extract and inner shell extract, respectively) [1].

### 3.3. Cell Viability Studies

In order to evaluate the possibility to use the optimal extract as new ingredient for different industries, cell viability studies were performed in intestinal (Caco-2 and HT29-MTX) and skin (HaCaT and HFF-1) cell lines. These cells are considered suitable in vitro models for testing the toxicity potential of substances or products intended for oral and dermatological use [58,59]. Caco-2 are morphological and functionally similar to enterocytes, while HT29-MTX are commonly employed for digestion and bioavailability studies of food compounds [60]. The results of the cell viability assay are summarized in Table 6.

According to the results shown in Table 6, *C. sativa* extract obtained by UAE did not cause a decrease in keratinocytes viability at a concentration of 0.1 µg/mL. However, at concentrations of 1, 10, and 100 µg/mL, cell viability was respectively 79.1%, 77.0%, and 80.4%, while at the concentration of 1000 µg/mL the cell viability was 68.0%. In this way, only the concentration of 0.1 µg/mL did not affect the cell viability.

Pinto et al. evaluated the effect on keratinocytes viability after exposure to chestnut bur extract obtained by maceration with a hydroalcoholic mixture at 40 °C for 30 min [61]. According to the authors, the extract did not decrease the cell viability between 0.1 and 100 μg/mL. Nevertheless, at a concentration of 1000 μg/mL, the cell viability decreased to 57.1%, which is in line with the present results [61]. More recently, Squillaci et al. studied the effect on keratinocytes viability after exposure to an aqueous extract of chestnut shells (in concentrations between 0.0004% and 0.5%) prepared in boiling water for 1 h, reporting that the extract below 0.01% has no significant toxic effect [1].

Regarding fibroblasts viability, for all tested concentrations the viability is 100%, without significant differences (*p* > 0.05) between concentrations. According to the literature, this is the first time that the effect of chestnut extracts in fibroblasts is reported.

In what concerns to intestinal cell lines, after exposure to the tested concentrations, both lines did not achieve a viability of 100%. Once again, to the best of our knowledge, this is the first time that the effects on intestinal cell lines after exposure to chestnut shells extract is reported. Recently, Cacciola et al. exposed six tumor cell lines (DU 145, PC-3, LNCaP, MDA-MB-231, MCF-7, and HepG2) and one normal prostate epithelial cell line (PNT2) to different concentrations of aqueous chestnut shells extract (1–100 µg/mL) [62]. The authors reported a decrease on cell viability for less than 50% (DU 145, LNCaP, and PNT2 cells) at the maximum concentration tested (100 μg/mL), which is in line with this study [62].

## 4. Conclusions

In this study, ultrasonic assisted technology was used for the extraction of phenolics and antioxidants from chestnut shells. The RSM was employed to optimize the independent variables, namely ultrasonic extraction temperature (°C) and extraction time (min). The optimal conditions that maximized the amount of phenolics and antioxidant activity of CSS extracts were determined as follows: 70 °C of temperature and 40 min of extraction time. The efficiency of ultrasound-assisted extraction was strongly influenced by the extraction variables, indicating that chestnut shells are a good source of important antioxidants. Under the optimal conditions, the extraction yield was 16%; the optimal extract showed a high content of phenolic compounds (393.1 mg GAE/g dw) as well as a high antioxidant activity ((DPPH, IC_50_ = 44.1 µg/mL; FRAP, IC_50_ = 32.0 µg/mL; ABTS, IC_50_ = 65.4 µg/mL). Indeed, a good scavenging capacity of O_2_●^–^ (IC_50_ = 14.1 µg/mL), HOCl (IC_50_ = 0.7 µg/mL), NO● (IC_50_ = 0.1 µg/mL) and ROO● (0.1) was observed. In what concerns to the bioactive compounds present, ellagic acid is the major one (40.4 µg/mg), followed by caffeic acid derivative (15.4 µg/mg) and epigallocatechin (15.3 µg/mg). The effect on skin and intestinal cell lines at different extract concentrations was also assessed by an MTT assay. According to the results obtained, there was no decrease in fibroblasts viability up to the maximum concentration tested (1000 µg/mL), while for keratinocytes the extract showed no adverse effects on the minimum concentration tested (0.1 µg/mL). The viability in intestinal cell lines did not achieved 100% for all tested concentrations. This study suggests for the first time the employment of UAE to extract bioactive compounds from CSS for different applications.

## Figures and Tables

**Table 1:** Experimental and predicted values of total phenolic compounds (TPC) (mg gallic acid equivalents (GAE)/g dw), FRAP (IC_50_ µg/mL), DPPH (IC_50_ µg/mL), and ABTS (IC_50_ µg/mL) of *C. sativa* shells extracts obtained by central composite design (CCD).

	*X*_2_ Time (min)	*X*_1_ Temp. (°C)	*Y*_1_ TPC (mg GAE/g dw)		Antioxidant Activity						Yield (%)
					*Y*_2_ FRAP (IC_50_ µg/mL)		*Y*_3_ DPPH (IC_50_ µg/mL)		*Y*_4_ ABTS (IC_50_ µg/mL)		
Exp.	X_1_	X_2_	Experimental ^a^	Predicted ^b^	Experimental ^a^	Predicted ^b^	Experimental ^a^	Predicted ^b^	Experimental ^a^	Predicted ^b^	
1	10	40	297.3 ± 7.6	308.5	39.8 ± 1.4	38.7	56.5 ± 0.1	53.4	66.4 ± 4.9	61.6	7.3 ± 1.2
2	40	40	350.5 ± 13.1	372.2	36.5 ± 2.8	33.9	59.9 ± 3.5	56.3	69.6 ± 1.5	66.0	11.5 ± 0.4
3	10	70	358.6 ± 9.0	337.2	36.1 ± 1.5	37.7	56.7 ± 2.1	58.4	64.4 ± 4.1	59.9	8.1 ± 0.1
4	40	70	393.1 ± 14.0	382.2	32.0 ± 1.5	32.1	44.1 ± 3.4	45.5	65.3 ± 5.6	62.1	16.1 ± 2.5
5	4	55	255.8 ± 4.7	263.1	45.3 ± 0.1	44.8	63.7 ± 5.2	64.3	50.5 ± 0.5	55.4	8.3 ± 0.7
6	46	55	347.6 ± 9.0	340.0	36.0 ± 2.1	37.5	56.1 ± 3.3	57.2	57.0 ± 3.5	60.2	10.3 ± 0.1
7	25	34	408.0 ± 20.3	384.8	28.7 ± 0.1	31.1	43.7 ± 3.2	48.1	64.7 ± 5.0	69.0	11.7 ± 1.6
8	25	76	389.4 ± 23.6	412.2	30.5 ± 1.4	29.1	46.7 ± 2.0	44.0	61.3 ± 2.3	65.1	13.4 ± 0.9
9	25	55	360.3 ± 8.4	356.0	36.2 ± 2.2	36.3	55.4 ± 1.9	57.2	87.7 ± 3.0	80.6	10.9 ± 1.5
10	25	55	336.0 ± 22.9	356.0	36.5 ± 0.7	36.3	51.5 ± 2.1	57.2	71.8 ± 0.1	80.6	8.5 ± 1.7
11	25	55	365.5 ± 9.9	356.0	35.4 ± 1.3	36.3	57.7 ± 1.1	57.2	72.6 ± 1.9	80.6	11.4 ± 6.7
12	25	55	355.1 ± 16.7	356.0	38.8 ± 1.5	36.3	58.8 ± 0.1	57.2	88.0 ± 3.0	80.6	8.6 ± 1.9
13	25	55	363.1 ± 9.6	356.0	34.7 ± 1.3	36.3	62.8 ± 4.3	57.2	83.0 ± 5.5	80.6	9.3 ± 0.8

^a,b^ Experimental values, performed in a random order and expressed as the average of triplicate determinations from different experiments (*n* = 3). IC_50_ = In vitro concentration required to decrease in 50% the reactivity of the studied reactive species.

**Table 2:** Model summary and analysis of variance (ANOVA) of TPC (mg GAE/g dw), FRAP (IC_50_ µg/mL), DPPH (IC_50_ µg/mL) and ABTS (IC_50_ µg/mL) of *C. sativa* shells extracts.

	Sum of Squares				Mean Squares				*F* Value				*p*-Value			
	*Y*_1_	*Y*_2_	*Y*_3_	*Y*_4_	*Y*_1_	*Y*_2_	*Y*_3_	*Y*_4_	*Y*_1_	*Y*_2_	*Y*_3_	*Y*_4_	*Y*_1_	*Y*_2_	*Y*_3_	*Y*_4_
Model	16,263.02	181.32	389.76	1144.33	3252.60	36.26	77.95	228.87	7.87	8.20	4.37	4.15	0.0086 *	0.0077 *	0.0399 **	0.0452 **
X_1_ min	5909.92	52.97	50.20	22.17	5909.92	52.97	50.20	22.17	14.30	11.98	2.81	0.40	0.0069 *	0.0105 **	0.1373	0.5464
X_2_ °C	753.57	4.01	16.66	15.37	753.57	4.01	16.66	15.37	1.82	0.91	0.93	0.28	0.2190	0.3725	0.3661	0.6141
X_1_.X_2_	87.32	0.15	63.38	1.21	87.32	0.15	63.38	1.21	0.21	0.03	3.55	0.02	0.6597	0.8611	0.1014	0.8865
X_1_.X_1_	5152.91	40.68	21.38	905.69	5152.91	40.68	21.38	905.69	12.47	9.20	1.20	16.41	0.0096 *	0.0190 **	0.3098	0.0049 *
X_2_.X_2_	3146.96	67.71	216.01	321.95	3146.96	67.71	216.01	321.95	7.61	15.31	12.11	5.83	0.0281	0.0058 *	0.0103	0.0464 *
Residual	2893.59	30.95	124.86	386.38	413.37	4.42	17.84	55.20								
Lack of fit	2336.95	20.87	55.47	133.42	778.98	6.96	18.49	44.47	5.60	2.76	1.07	0.70	0.0648	0.1757	0.4571	0.5978
Pure error	556.64	10.08	69.40	252.96	139.16	2.52	17.35	63.24								
Total	19,156.61	212.27	514.63	1530.71												

*R*^2^*prev* (*Y*_1_) = 0.8490; *R*^2^ *adj* (*Y*_1_) = 0.7411; *Ratio* = 10.80; *R*^2^ *prev* (*Y*_2_) = 0.8542; *R*^2^ *adj* (*Y*_2_) = 0.7500; *Ratio* = 11.00; *R*^2^ *prev* (*Y*_3_) = 0.7574; *R*^2^ *adj* (*Y*_3_) = 0.5841; *Ratio* = 7.05; *R*^2^ *prev* (*Y*_4_) = 0.7476; *R*^2^ *adj* (*Y*_4_) = 0.5673; *Ratio* =4.99; *R*^2^ *prev* (*Y*_4_) = 0.7476; *R*^2^ *adj* (*Y*_4_) = 0.5673; *Ratio* =4.99. * significance at *p* < 0.01; ** significance at *p* < 0.05.

**Table 3:** TPC and antioxidant activity evaluated by TPC, DPPH, ABTS and FRAP assays of the optimal extract of *C. sativa shells* (70 °C/40 min).

	TPC (mg GAE/g dw)	DPPH (IC_50_; µg/mL)	ABTS (IC_50_; µg/mL)	FRAP (IC_50_; µg/mL)
Experimental value ^a^	393.1 ± 14.0	44.1 ± 3.4	65.4 ± 3.5	32.0 ± 1.5
Predicted value	382.3	45.5	62.1	32.1
*p* ^b^	0.066	0.414	0.226	0.499

IC_50_ = In vitro concentration required to decrease in 50% the reactivity of the studied reactive species in the tested media (mean ± standard error of the mean). ^a^ Results are expressed as mean ± standard deviation (*n* = 3). ^b^ indicates significant differences (*p* < 0.05).

**Table 4:** Superoxide anion radical (O_2_●^–^), hypochlorous acid (HOCl), peroxyl radical (ROO●) and nitric oxide (NO●^–^) scavenging capacities of optimal *C. sativa* shells extract. Values are expressed as mean ± standard deviation (*n* = 4).

	ROS			RNS
	IC_50_ (µg/mL)		Trolox Equivalents (µmol TE/mg dw)	IC_50_ (µg/mL)
	O_2_●^–^	HOCl	ROO●	NO●^–^
*C. sativa* shell extract	14.1 ± 0.5	0.7 ± 0.0	0.3 ± 0.0	0.1 ± 0.0
Positive controls				
Gallic acid	5.2 ± 0.3	1.3 ± 0.1	6.0 ± 0.2	0.2 ± 0.0
Catechin	49.0 ± 1.3	0.2 ± 0.0	46.4 ± 0.2	1.0 ± 0.0

IC_50_ = In vitro concentration required to decrease in 50% the reactivity of the studied reactive species in the tested media (mean ± standard error of the mean).

**Table 5:** Identification and quantification of the phenolic compounds from the optimal extract by LC-MS analysis.

No.	Tr (min)	[M – H]^−^	Fragments	Identification	Amount (µg/mg dw)
1	10.5	289	-	Catechin/epicatechin	7.4 ± 0.1
2	12.4	301	284 257 229 2213 201 185 173	Ellagic acid	40.4 ± 0.1
3	13.7	1153	-	Tetrameric PAC	1.0 ± 0.1
4	14.0	305	179	Epigallocatechin	15.3 ± 0.1
5	19.2	577	269	Apigenin-7-*O*-rutinoside	0.8 ± 0.0
6	19.3	577	-	Trimeric PAC	4.2 ± 0.1
7	20.2	593	285 257 241 217 199 175	Luteolin-7-*O*-rutinoside	1.1 ± 0.1
8	22.6	589	-	Trimeric PAC	1.1 ± 0.1
9	28.7	341	179 161 143	Caffeic acid derivative	15.4 ± 0.1
10	45	1431	-	Procyanidin polimers	11.1 ± 0.1

**Table 6:** Effects of optimal extract exposure on the viability of Caco-2, HT29-MTX, HaCaT, and HFF-1 cells at different concentrations, as measured by the MTT assay.

	Cell Lines			
Concentration (µg/mL)	Caco-2	HT29-MTX	HaCaT	HFF-1
Medium	100.0 ± 14.5	100.0 ± 18.8	101.0 ± 3.4	100.0 ± 18.0
Triton X-100	0.0 ± 0.0	0.0 ± 0.0	0.0 ± 1.0	0.0 ± 4.1
0.1	68.8 ± 10.2	67.0 ± 10.4 ^a,b^	97.8 ± 17.8 ^a^	105.6 ± 1.6
1	55.6 ± 6.1	74.0 ± 14.3 ^a^	79.1 ± 14.6 ^b^	98.0 ± 18.3
10	66.3 ± 3.1	35.5 ± 6.7 ^c^	77.0 ± 9.6 ^b^	117.2 ± 20.8
100	79.2 ± 15.5	43.9 ± 3.8 ^b,c^	80.4 ± 10.7 ^b^	118.0 ± 5.8
1000	70.4 ± 5.7	65.3 ± 1.0 ^a,b^	68.0 ± 7.0 ^b^	87.7 ± 7.7

Values are expressed as mean ± standard deviation (*n* = 4). Different letters (a,b,c) in the same cell line represent significant differences (*p* < 0.05) between different concentrations of extract, according to Tukey’s HSD test.
